# Human Cardiac Organoids for Modeling Genetic Cardiomyopathy

**DOI:** 10.3390/cells9071733

**Published:** 2020-07-20

**Authors:** Michele Filippo Buono, Lisa von Boehmer, Jaan Strang, Simon P. Hoerstrup, Maximilian Y. Emmert, Bramasta Nugraha

**Affiliations:** 1 Institute for Regenerative Medicine, University of Zurich, 8952 Schlieren, Switzerland; michele.buono@me.com (M.F.B.); lisa.vonboehmer@uzh.ch (L.v.B.); simon.hoerstrup@irem.uzh.ch (S.P.H.); 2 Zurich University of Applied Sciences, 8820 Wädenswil, Switzerland; strang.jaan@gmail.com; 3 Wyss Translational Center Zurich, 8006 Zurich, Switzerland; 4 Department of Cardiovascular Surgery, University Hospital Zurich, 8091 Zurich, Switzerland; 5 Department of Cardiovascular Surgery, Charité Universitätsmedizin Berlin, 10117 Berlin, Germany

**Keywords:** hiPSCs, cardiomyocytes, differentiation, 3D culture, organoids, in vitro disease modeling, personalized medicine

## Abstract

Genetic cardiomyopathies are characterized by changes in the function and structure of the myocardium. The development of a novel in vitro model could help to better emulate healthy and diseased human heart conditions and may improve the understanding of disease mechanisms. In this study, for the first time, we demonstrated the generation of cardiac organoids using a triculture approach of human induced pluripotent stem-cell-derived cardiomyocytes (hiPS-CMs)—from healthy subjects and cardiomyopathy patients—human cardiac microvascular endothelial cells (HCMECs) and human cardiac fibroblasts (HCFs). We assessed the organoids’ suitability as a 3D cellular model for the representation of phenotypical features of healthy and cardiomyopathic hearts. We observed clear differences in structure and beating behavior between the organoid groups, depending on the type of hiPS-CMs (healthy versus cardiomyopathic) used. Organoids may thus prove a promising tool for the design and testing of patient-specific treatments as well as provide a platform for safer and more efficacious drug development.

## 1. Introduction

Presently, cardiovascular diseases (CVD) represent the main cause of death worldwide [1]. Cardiomyopathies (nongenetic and genetic)—a set of conditions that compromise the contractile activity of the heart in affected patients—represent a highly relevant subset of CVD. The disease mechanisms in genetic cardiomyopathy are extremely variable, making it difficult to establish a universal efficacious treatment and causing a tremendous cost burden on healthcare systems [2].

The development of novel drugs and treatments is a long and expensive process, often involving years or decades of research, potentially leading to ineffectual results [3,4]. Indeed, the efficacy and safety of novel medications are first evaluated during a preclinical drug discovery process which is typically performed in 2D cell cultures and subsequently in animal models. Unfortunately, both models fail in adequately representing human physiological conditions and incur the risk of novel candidate failure [5,6,7]. Adverse events at a late stage of clinical trials can cause substantial temporal and financial loss [8]. To overcome these limitations and to enhance the understanding of biological mechanisms during the preclinical stage of the drug discovery process, a new concept based on culturing cells in three dimensions (3D) has been suggested [9]. It is believed that 3D human organoid models will eventually promote better understanding of human disease mechanisms, and help direct future treatments towards personalized medicine [10,11,12,13].

The advances in human induced pluripotent stem cell (hiPSC) technology allow for in vitro disease modeling and tissue regeneration research geared towards patient-specific treatments. They also give rise to the possibility of developing functional human organoids to emulate a multitude of organ systems and conditions more easily [14,15]. One key feature of hiPSCs is the capacity of the cells to retain the patient genotype from which they were derived [16]. Lan et al. showed that cardiomyocytes (CMs) differentiated from hiPSCs derived from a hypertrophic cardiomyopathy (HCM) patient containing a gene mutation encoding for myosin heavy chain 7 (MYH7) retained the mutation and displayed the HCM phenotype, which is characterized by cellular hypertrophy and abnormal calcium handling activity as evidenced by irregular beating/arrhythmia [17]. We narrowed our research focus on a missense mutation of MYH7 in exon 19 where a single point mutation (SNP) causes an amino acid change from Arginine into Glutamine, referred to as Arg719Gln or R719Q-MYH7 mutation. Despite being annotated as a benign mutation, this SNP has been known to play a role in the onset of HCM causing sudden cardiac death [18,19].

Despite well-established chemically-defined methods to efficiently differentiate hiPSCs into high quality cardiomyocytes (CMs), only few studies have so far investigated cardiac organoids for mimicking the human heart and its functions [20,21,22,23]. Given the advances in reprogramming somatic cells to obtain hiPSC lines reflecting patient-specific genome profiles and in differentiating hiPSCs into cardiomyocytes (hiPSC-CMs) resembling the corresponding cardiac phenotypes, a multicellular 3D bioengineered tissue construct strategy is deemed a promising approach for modeling the human heart in vitro [24,25,26]. However, the myocardium also contains nonmyocyte cells, which play essential roles in cardiac physiology and function. Cardiac fibroblasts and cardiac endothelial cells represent the majority of nonmyocytes present in the adult heart [23,25]. Cardiac fibroblasts contribute to tissue maintenance and remodeling through the synthesis of extracellular matrix (ECM) and directly affect the electrical properties of the myocardium. Endothelial cells (ECs) regulate the contractile state, survival, metabolism and rhythmicity of CMs through autocrine and paracrine signaling [26,27]. The combination of these three cells types, therefore, may have the potential to significantly improve the cardiac research field through the development of human cardiac organoids. The latter can mimic various heart developmental stages, and can be used not only as a reliable platform for drug screening but also as a tool to imitate patient-specific cardiac conditions and assess the effectiveness of tailored personalized treatments [12].

In this study, as a first step, we compared two chemically defined protocols for the differentiation of hiPSCs into CMs. Two commercially available hiPSC lines, one obtained from a healthy patient and one from an HCM-patient carrying an MYH7 mutation, were differentiated into CMs. hiPSC-CM quality was assessed by the evaluation of cell morphology and beating intensity. The better-suited protocol was chosen based on the quality of the hiPSC-CMs at harvesting day. The differentiated cells were subsequently characterized in terms of cell population purity using flow cytometry and in terms of differentiation status using quantitative gene expression analysis. Further optimization of the culturing conditions for the creation of cardiac organoids containing CMs and nonmyocytes was performed. We obtained highly contractile cardiac organoids consisting of hiPSC-CMs, human cardiac microvascular endothelial cells (HCMECs) and human cardiac fibroblasts (HCFs) by seeding a mix of corresponding single cell suspensions. The organoids containing either of the two hiPSC-derived CMs (healthy and cardiomyopathy-associated) were then compared in terms of cell distribution, structural integrity, differentiation status and calcium activity, in order to assess their capability of representing the donors’ phenotypic features.

## 2. Materials and Methods

Materials were purchased from Sigma Aldrich, unless stated otherwise.

### 2.1. Cell Culture

#### 2.1.1. Human Cardiac Microvascular Endothelial Cells

Human Cardiac Microvascular Endothelial Cells (HCMECs) are primary cells isolated from human heart tissue. HCMECs were purchased from ScienCell Research Laboratories (Carlsbad, USA) and cultured in cell culture flasks precoated with 2 µg/cm^2^ Bovine Plasma Fibronectin (BPF) (#8248) diluted in Endothelial Cell Medium (ECM) (#1001). The ECM was supplemented with 5% Fetal Bovine Serum (#0025), 1% Endothelial Cell Growth Supplement (ECGS) (#1052) and 1% Penicillin-Streptomycin solution (#0503).

#### 2.1.2. Human Cardiac Fibroblast

Human Cardiac Fibroblasts (HCFs) are primary cells isolated from healthy adult human heart tissue. Cryopreserved HCFs were purchased from Cell Application (San Diego, CA, USA) and cultured in HCF Growth Medium (#316K-500).

#### 2.1.3. Cor.4U Cardiomyocytes

Cor.4U Cardiomyocytes (Cor.4U-CMs) are commercially available human embryonic stem cell-derived cardiomyocytes (hiPSC-CMs) containing a mix of 60% ventricular, 20% atrial, and 20% nodal cells. One frozen cryovial (1 × 10^6^ cells) was purchased from NCardia Stem Cell Expert (Leiden, the Netherlands). Cor.4U-CMs were cultured as recommended by the manufacturer. Briefly, 1 × 10^6^ Cor.4U-CMs were thawed and plated in a 25 cm^2^ culture flask (T25) (TPP Techno Plastic Products AG, Trasadingen, Switzerland), precoated with 10 µg/mL Fibronectin (Sigma-Aldrich, #F1141) in Dulbecco’s Phosphate Buffered Saline (DPBS) with Ca^2+^ and Mg^2+^ (Gibco, #14040-133) in Complete Culture Medium (#Ax-M-HC250), supplemented with 2 µg/mL Puromycin (#Ax-M-PO-05). After 24 h incubation, the medium was changed and the flask was kept in culture at 37 °C, 5% CO_2_. Forty-eight hours post-thawing, the Cor.4U-CMs were dissociated using 0.05% Trypsin EDTA (Life Technologies, #15400, Zug, Switzerland) and immediately used to generate triculture organoids.

#### 2.1.4. Human Induced Pluripotent Stem Cell Lines

Two commercially available hiPSC lines were purchased from the European Bank of Induced Pluripotent Stem Cells (EBiSC, Salisbury, UK). The Wellcome Trust Sanger Institute (WTSIi020-A) line was derived from a healthy donor and the Uniklinik Köln (UKKi025-A) line originated from a hypertrophic cardiomyopathy donor (see Table 1). All cell culture reagents for hiPSCs were purchased from Stem Cell Technologies (Cologne, Germany), unless stated otherwise.

Both cell lines were cultured in a six-well plate (TPP, Switzerland), precoated with 10 µg/mL Vitronectin^XF^ (#07180) in CellAdhere dilution buffer (#07183). On seeding day, each cryovial was thawed and seeded into two wells in 2 mL/well mTeSR1 complete medium (#85850) supplemented with 10 μM Y-27632 (#72304).The latter is a ROCK pathway inhibitor that significantly enhances the recovery of frozen stocks and improves hiPSCs attachment and growth in culture vessels. After 24 h at 37 °C with 5% CO_2_, the ROCKi medium was replaced with mTeSR1 complete medium, which was replenished daily until about 70% confluency. Subculturing with a split ratio of 1:6 was done when cell colonies presented as compact clusters with well-defined edges by using a nonenzymatic reagent, ReLeSR (#05872). Cryopreservation was done using animal component-free medium, CryoStor CS10 (#07930), containing 10% DMSO.

### 2.2. Cardiomyocyte Differentiation

#### 2.2.1. STEMdiff Differentiation Protocol

WTSIi020-A (passage no. ≥38) and UKKi025-A (passage no. ≥41) were cultured in mTeSR1 medium (StemCell Technologies, Vancouver, BC, Canada) in Vitronectin^XF^-coated 12-well plates until 95% confluence. The STEMdiff Cardiomyocyte Differentiation Kit (StemCell Technologies, #05010) was used to differentiate hiPSCs into CMs in eight days by following the manufacturer’s protocol. From day 8 to 15, the obtained hiPSC-CMs (WTSI-CMs and UKK-CMs) were maintained in culture, using the STEMdiff Maintenance Kit (#05020), as recommended by the supplier. On day 15, cell harvesting was performed by dissociating the hiPSC-CMs, using the STEMdiff Cardiomyocyte Dissociation Kit (#05025) and a fraction of the cells was cryopreserved in STEMdiff Cardiomyocyte Freezing Medium (#05030) at a cell density of 1 × 10^6^ cells/mL. To reduce stress due to cell thawing and to improve hiPSC-CMs recovery, STEMdiff Cardiomyocyte Support Medium (#05027) was applied for 24 h after thawing and then replaced with STEMdiff Cardiomyocyte Maintenance Medium for long-term culture.

#### 2.2.2. CDM3 Differentiation Protocol

WTSIi020-A (passage no. ≥38) and UKKi025-A (passage no. ≥41) were cultured and expanded in six-well plates. At 70% confluence, the cells were split at a 1:4 ratio and replated in Vitronectin^XF^-coated 12-well plates until about 85% confluence. CM differentiation was performed as described by Burridge et al. 2014 [21]. Briefly, the cell medium was changed to CDM3, consisting of RPMI 1640 (11875, Life Technologies) supplemented with 500 μg/mL Oryza sativa-derived recombinant human albumin (A0237, Sigma-Aldrich, 75 mg/mL stock solution in WFI H2O, stored at −20 °C) and 213 μg/mL L-ascorbic acid 2-phosphate (Sigma-Aldrich, 64 mg/mL stock solution in WFI H2O, stored at −20 °C). The medium was changed every other day (48 h). For the first 48 h post cell replating, CDM3 was supplemented with 6 μM CHIR99021 (LC Laboratories). On day 2, the medium was changed to CDM3 supplemented with 2 μM Wnt-C59 (Selleck Chemicals). The medium was then again changed on day 4, and every other day until day 15 using CDM3. Contracting cells were observed from day 7 onwards. On day 15, both hiPSC-derived cardiomyocyte lines (WTSIi020-A-CMs and UKKi025-A-CMs) were dissociated with TrypLE Express (1×) containing phenol red (ThermoFisher Scientific, #12605010) for 10 min at 37 °C and used for gene expression analysis (RT-qPCR), cell population analysis (FACS) and triculture organoid formation. Day 15 dissociated cells were counted using a LUNA Automated Cell Counter (Logos Biosystems, Villeneuve-d’Ascq, France) and a fraction of the cells was cryopreserved at 1 × 10^6^ cells per vial in CryoStor^®^ CS10.

### 2.3. Flow Cytometry

WTSIi020-A-CMs and UKKi025-A-CMs were harvested on day 0 and 15 to evaluate the efficacy of the differentiation protocol (CDM3) in producing hiPSC-CMs. The dissociated cells were directly transferred to flow cytometry tubes (BD Biosciences) and stained with a live/dead discriminator using 1:1000 Zombie Aqua Dye (BioLegend, #423101). Samples were fixed with 4% PFA for 15 min and stored in FACs buffer, containing 2% FBS, 5mM EDTA, 0.01% Sodium Azide in PBS, at 4 °C for later use. Before flow cytometry analysis, samples were permeabilized using a blocking buffer containing 20% FBS, 3% BSA, 0.2% Saponin in PBS for 10 min at RT. They were then stained using an antibody solution containing 1% BSA and 0.1% Saponin in PBS, 1:600 mouse monoclonal IgG1 TNNT2 (cardiac troponin T, clone 1G1, #MA5-17192) and rabbit monoclonal IgG OCT4 (Octamer-binding transcription factor 4, clone T.631.9, #MA5-14845) primary antibodies (ThermoFisher Scientific, USA) for 1 h in the dark at RT. Secondary antibody staining was performed with 1:600 dilutions of Alexa Fluor 488 and Alexa Fluor 568 secondary antibodies (Life Technologies) for 30 min at RT. Samples were analyzed using LSRFortessa (BD Biosciences) and FACSDiva Software. The resulting data was analyzed using FlowJo v10 (TreeStar).

### 2.4. Real-Time Quantitative PCR Analysis

WTSIi020-A-CMs and UKKi025-A-CMs were collected at two differentiation time points, day 0 as a control for pluripotent markers and day 15 to check the efficiency of the CM differentiation protocol. Cor.4U-CMs (1 × 10^6^) were also sampled and used as a positive control for cardiac markers. Samples were lysed with RLT buffer (QIAGEN) and stored at −80 °C before proceeding with mRNA extraction. RNA samples were obtained using RNeasy Mini Kit (QIAGEN, #74104). 100 ng RNA per sample were reverse transcribed to 20 µL cDNA (1:1 reaction) using iScript Reverse Transcription Supermix (Bio-Rad, #1708841). Quantitative gene expression analysis was performed using TaqMan Fast Universal PCR Master Mix (2×), no AmpErase UNG (Applied Biosystems, #4366073), TaqMan Gene Expression Assays (listed in Table 2) and an Applied Biosystems 7500 Fast Real-Time qPCR System. All reactions were performed in triplicate and normalized using the 18S endogenous housekeeping gene. Relative quantification of gene expression was performed using the ∆∆C_t_ method, where 2^−ΔΔCt^ was calculated to obtain the expression fold change value of each gene.

### 2.5. Organoid Formation Technique

Triculture organoids were created by combining hiPSC-CMs (Cor.4U-CMs or WTSI-CMs or UKK-CMs) with HCMECs and HCFs in a single-cell suspension at a physiological cell ratio of 3:5:2, as suggested by Devalla and Passier [28]. Greiner HLA Terasaki 60-well plates (Sigma-Aldrich, #M6062) were used to create the organoids utilizing a modified hanging drop protocol [5,29]. Approximately 100,000 cells in 20 µL cell suspension were pipetted into each well. Organoids were harvested after three days and transferred to Poly-L-Lysine (Sigma-Aldrich, #P4707) precoated 12-well plates for long-term culture (21 days).

### 2.6. Cell and Organoid Immunofluorescence Staining

Cells were fixed with 4% PFA (Sigma) for 20 min at room temperature. They were permeabilized with a solution of 0.2% saponin/20% FBS/3% BSA, and further stained with primary and secondary antibodies.

Organoids were harvested and immunofluorescence stained using the Visikol Histo Organoid Protocol. Briefly, organoids were transferred to 12-well plates and fixed overnight (≤16 h) in 4% PFA (Sigma Aldrich, Buchs, Switzerland). After fixation, they were transferred into a 0.05% sodium azide solution in PBS for long-term storage before processing.

All antibodies used were purchased from ThermoFisher Scientific Zurich, Switzerland, and further diluted in Visikol Antibody Buffer (Hampton, NJ, USA). Primary antibodies used were as follows: mouse antihuman alpha sarcomeric actinin (1:300, #MA122863), goat antihuman cardiac troponin (TNNT2, 1:300, #PA1-86820), rabbit antihuman vimentin (VIM, 1:300, #MA5-16409) and mouse antihuman vascular cadherin (VeCADH, 1:300, #14-1449-82). DAPI solution (1 mg/mL, #62248) was used as a nuclear label, diluted 500-fold in Visikol Antibody Buffer, and incubated for 5 min at 37 °C between the application of the primary and secondary antibodies. Secondary antibodies used were as follows: rabbit anti-goat Alexa Fluor 647 (#A27018), goat anti-rabbit Alexa Fluor 488 (#A11034) and goat anti-mouse Alexa Fluor 568 (#A11004). Images were taken with a Leica SP8 confocal microscopy system.

### 2.7. Calcium Activity Monitoring Assay

Calcium activity in highly contractile cardiac organoids was assessed using the EarlyTox Cardiotoxicity Kit (Molecular Devices). The kit contains a dye which binds to calcium ions as they enter the cell cytoplasm, allowing for the measurement of changes in calcium concentrations. Briefly, Cor-Oids, WTS-Oids and UKK-Oids were transferred to eight-well µ-slides (Ibidi—cells in focus, #80824), containing 100 μL of medium and 100 μL of prewarmed calcium dye per well, and incubated for 2 h at 37 °C. The calcium activity within the organoids was observed using widefield microscopy and recorded for 90 s. The beating pattern of each organoid type was analyzed and plotted using Fiji- ImageJ v2.0.0. The beating rates per minute (bpm) were calculated in Fiji- ImageJ v2.0.0 using the BAR plug-in, which determines the peaks in the plotted organoid beating curves.

### 2.8. Statistical Analysis

**FACS**: the flow cytometry standard files obtained from the FACS analysis were processed using FlowJo. Firstly, gating of the forward (FCS) versus side scatter (SCC) was determined. To determine the live cell population, the singlets and zombie aqua dye negative events were selected. The Median Fluorescence Intensity (MFI) over the differentiation time (day 0 vs day 15) in TNNT2 and OCT4 cells was recorded, plotted and compared by a two-way ANOVA test using the GraphPad Prism 8 software. All data are presented as mean ± standard error of the mean (SEM). A *p*-value ≤0.01 was deemed statistically significant.

**qPCR**: 18S was selected as the housekeeping gene and amplification data were used to quantify the expression of the genes of interest. Relative expression values (2^−ΔΔCt^) were calculated using Excel (Microsoft). The unpaired t-test (two-tail) was used to compare the different time points for each gene investigated (day 0 vs day 15). All data are presented as mean ± standard error of the mean (SEM). A *p*-value ≤ 0.01 was deemed statistically significant.

## 3. Results

### 3.1. Differentiation of hiPSCs into Cardiomyocytes

**Figure 1:**
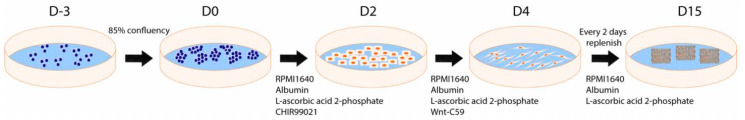
Illustration of hiPSC differentiation with Chemically-Defined Medium 3 (CDM3) protocol adapted from Burridge et al. [21].

Cardiomyocytes were differentiated from hiPSCs, comparing two differentiation protocols, STEMdiff and CDM3 adapted from [21], using two commercially available patient-derived hiPSC lines: WTSIi020-A and UKKi025-A, corresponding to a healthy donor and an HCM patient, respectively. Various differentiation cell batches were produced to optimize the protocol for our study (see Table 3). Cardiac differentiation was induced in both lines with the results showing that the production of hiPSC-CMs using the STEMdiff protocol exhibited high variability, while the CDM3 protocol was found to be more stable and effective, especially when started at a cell passage number >40 and at higher than 85% cell confluency (Figure 1 and Table 3). The quality of our hiPSC-CMs was assessed by visual inspection of their morphology and beating intensity at harvesting day. Both WTSIi020-A derived CMs (WTSI-CMs) and UKKi025-A derived CMs (UKK-CMs) obtained from five differentiation batches were frozen and banked to assess their survival after thawing. WTSI-CMs and UKK-CMs obtained using the STEMdiff protocol showed low cell recovery post-thawing (10–20% survival), while the ones differentiated according to the CDM3 protocol displayed better cell recovery post-thawing (40–50%), when using the freezing media as suggested by the protocols. To improve recovery post-thawing, another freezing medium, normally used for hiPSC cryopreservation (CryoStor), was tested, which improved the cell recovery of CMs differentiated in CDM3 up to 60–70% post-thawing.

### 3.2. Characterization of hiPSC-CMs

**Figure 2:**
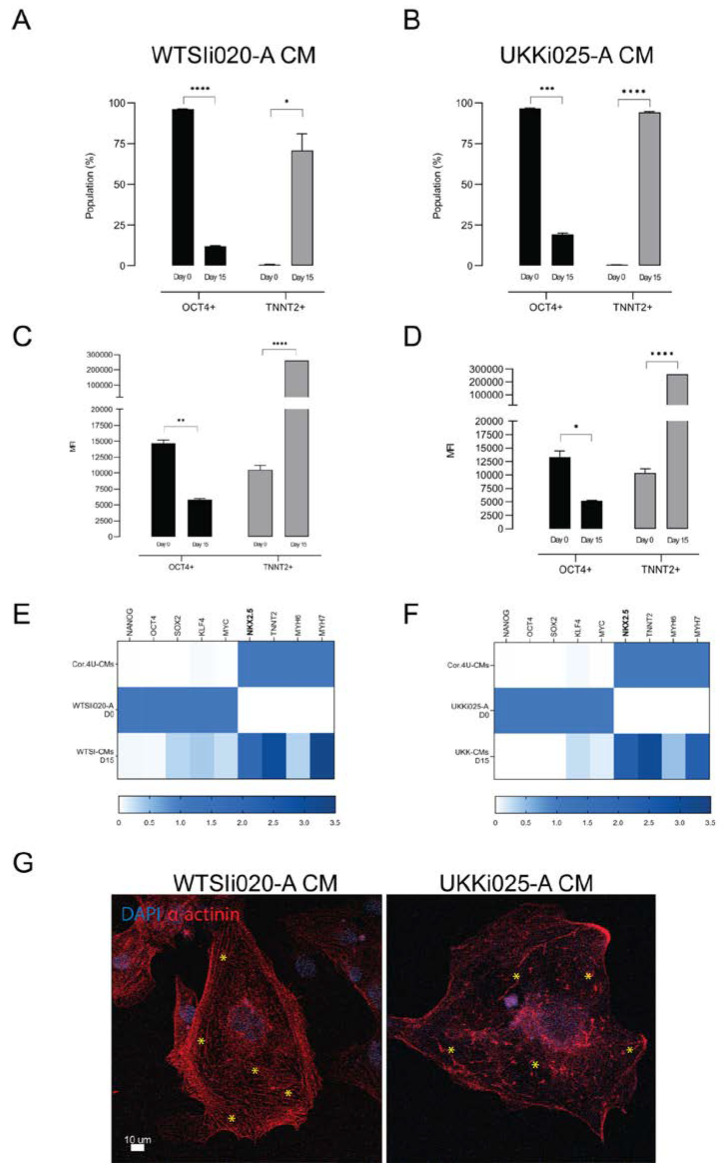
Characterization of cardiomyocytes derived from WTSIi020-A and UKKi025-A. (**A**,**B**) Flow cytometry analysis of cells positive for TNNT2 and OCT4 at the beginning (day 0) and the end (day 15) of differentiation in CDM3. (**C**,**D**) Median fluorescence intensities of TNNT2^+^ and OCT4^+^ populations at day 0 and 15 of the CDM3 protocol. n = 3 biological replicates. (**E**,**F**) Heatmap plots representing RT-qPCR for markers of pluripotency (NANOG, OCT4, SOX2, KLF4, MYC), cardiac progenitors (NKX2.5) and cardiomyocytes (TNNT2, MHY6, MHY7) on day 0 and day 15. n = 3 independent biological replicates performed in technical triplicates. (**G**) Immunofluorescent staining images of cardiomyocytes derived from WTSIi020-A and UKKi025-A. WTSIi020-A CMs show clear formation of immature sarcomeric-like structure while UKKi025-A CMs display a disrupted structure and rather enlarged cell morphology (marked with *). Significance level **A**: **** *p*_oct4_ < 0.001; * *p*_tnnt2_ < 0.023; **B**: *** *p*_oct4_ = 0.0002; **** *p*_tnnt2_ < 0.0001; **C**: ** *p*_oct4_ = 0.0015; **** *p*_tnnt2_ < 0.0001; **D**: * *p*_oct4_ = 0.0142; **** *p*_tnnt2_ < 0.0001; **E**: *p* ≤ 0.0001; **F**: *p* ≤ 0.0002.

The robustness of the selected differentiation protocol (CDM3) was assessed in terms of proportion of differentiated to undifferentiated cells in the population, by performing flow cytometry using cardiac and pluripotency markers. Cardiac-specific troponin (TNNT2) was used to specifically label differentiated CMs [21,30], and octamer-binding transcription factor 4 (OCT4) [31] was used to check for the presence of undifferentiated hiPSCs. Flow cytometry analysis was performed on sampled cells from the fifth differentiation batch on day 0 and day 15. The efficiency of the CDM3 protocol for both hiPSC lines was demonstrated by the finding that 75% of WTSI-derived cells and 97% of UKK-derived cells were positive for cardiac troponin at day 15 (Figure 2A,B). The fluorescence intensities of TNNT2^+^ and OCT4^+^ cells on day 0 and 15 were measured and the median fluorescence intensities (MFI) were calculated (Figure 2C,D). The data showed a decrease in the number of OCT4^+^ cells from day 0 to day 15, while a significant increase in TNNT2^+^ cells occurred over time, which underlines the robustness of the differentiation protocol and indicates high cell purity for both WTSI-CM and UKK-CM populations.

The fifth batch cells were also sampled and examined for pluripotent and cardiac gene patterns (Day 0 vs Day 15), comparing them with a commercially available cardiomyocyte line, Cor.4U-CMs, by real-time quantitative PCR (RT-qPCR) (Figure 2E,F). In concordance with previously reported patterns [21,32], the pluripotency markers Nanog Homeobox Protein (NANOG), OCT4 and SRY (Sex determining Region Y)-box 2 (SOX2) were found to have been down-regulated on day 15 when compared to day 0, meaning that the pluripotent state, normally maintained in undifferentiated cells by a complex network made of these transcription factors, was diminished [31]. The expression level of tumorigenic markers Kruppel Like Factor 4 (KLF4) and V-Myc Avian Myelocytomatosis Viral Oncogene Homolog (MYC) [33] resulted in a significant decrease at day 15, indicating an absence of carcinogenicity in the differentiated populations [34]. As expected, cardiac-specific markers such as NKX2.5, TNNT2, MYH6 and MYH7 were found completely absent on day 0 while highly expressed on day 15. Surprisingly, the cardiac progenitor NK2 Homeobox 5 (NKX2.5), cardiac troponin T2 (TNNT2), embryonal myosin heavy chain (MYH6) and adult myosin heavy chain (MYH7) were found to have similar expression levels in both hiPSC-CM populations. This could be related to the cardiomyocyte maturity status. Indeed, NKX2.5 and MHY6 are normally highly expressed during CM differentiation and myofilament establishment, the latter of which introduces contractile capacity in the cells [35]. The contraction-relaxation cycle promotes the maturation of fetal/immature CMs [28,36]. Mature CMs should display a high level of MYH7 and low or no MYH6 expression. TNNT2 is expressed in both immature and mature CMs, with the difference being that in mature cells its expression level is somewhat constant and stable [37]. The above-mentioned gene expression results indicate that our day 15 WTSI-CMs and UKK-CMs, differentiated in CDM3 medium, were still immature.

In concordance with the gene expression results, immunofluorescent staining images (Figure 2G) showed clear formation of immature sarcomeric-like structures in WTSIi020-A CM, while UKKi025-A CM exhibited discontinued actin filament fibers and a rather enlarged cell morphology. The beating patterns of these two types of cardiomyocytes also differed, in that the UKKi025-A CMs (Supplementary Video S1B) displayed arrhythmic beating as opposed to the WTSIi020-A CMs (Supplementary Video S1A).

### 3.3. Generation of Triculture Cardiac Organoids

In a next step, highly contractile cardiac organoids—composed of hiPSC-CMs, primary human cardiac microvascular endothelial cells (HCMECs) and primary human cardiac fibroblasts (HCFs)—were generated, using Terasaki plates in a modified hanging drop approach [5,29]. 25 µL of the cell mixture were pipetted onto each Terasaki well, inverted and incubated for at least three days, until organoids were formed. The cell seeding density was optimized at 100,000 cells per organoid and the cell ratio was 3:5:2, i.e., three parts CMs, five parts endothelial cells and two parts fibroblasts, mimicking adult human heart tissue, as previously described by Devalla and Passier [28]. 

#### 3.3.1. Proof of Concept Experiment: Gold Standard Cor-Oids

To validate the triculture approach, the organoid formation concept was initially tested and confirmed by generating contractile cardiac organoids containing commercially available hiPSC-CMs. Cor.4U-CMs were tricultured together with the HCMECs and HCFs. The Cor.4U-based organoids (referred to as Cor-Oids) were chosen as a positive control for comparison to organoids containing our differentiated hiPSC-CMs.

**Figure 3:**
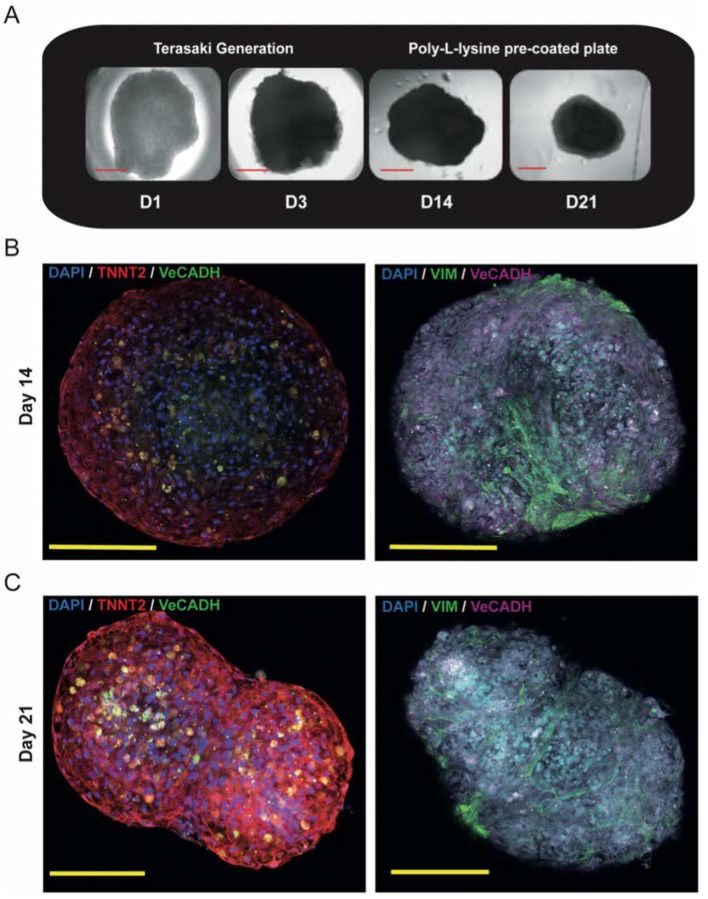
Modelling Adult Human Heart Organoids. (**A**) Generation of contractile cardiac organoids (Cor-Oids) containing Cor.4U-CMs, HCMECs and HCF at 3:5:2 cell ratio using Terasaki plates showed the formation of compact ball-like shapes after 21 days of culture. (**B**) Immunofluorescence staining images of Cor-Oids after 14 days of culture, labelled with cardiac-specific troponin (TNNT2), vascular cadherin (VeCADH) and vimentin (VIM), showing organoids with a lack of defined cardiac structures (T-tubules and sarcomeres). (**C**) Immunofluorescence staining images of Cor-Oids after 21 days of culture, labelled with cardiac-specific troponin (TNNT2), vascular cadherin (VeCADH) and vimentin (VIM), showing CMs migration toward the organoid core, promoting the formation of cardiac filament-like structures. Scale bars = 200 µm.

On day 3 after triculture *seeding*, the Cor-Oids were harvested from the Terasaki plates and transferred onto Poly-L-Lysine pre-coated plates (Figure 3A) to prevent complete attachment and spreading on the bottom of the well plate. They were then maintained in culture until day 21 (D21), when they showed an increased contraction activity compared to the harvesting day (D3). On day 21, the Cor-Oids shrunk in dimension, assuming a compact and well-defined spherical shape, showing significantly increased beating intensities.

The cell distribution and the structural integrity of the Cor-Oids were analyzed and confirmed via immunofluorescence staining for cell-specific markers after 14 and 21 days post-seeding, using TNNT2, VeCADH and VIM as specific markers for CMs, HCMECs and HCFs, respectively. All expected cell types were positively identified within the organoids. The CMs showed a preference to localize at the organoids’ perimeter, while HCMECs and HCFs were localized predominantly in the inner part, on day 14 (Figure 3B).

Interestingly, a more prominent CM migration to the organoids’ core was observed in day 21 Cor-Oids (Figure 3C), implying that one additional week of culture appears to be advantageous for cell distribution and migration. A more evenly distributed cell pattern was evident, and the CMs (red) formed an interlaced filament-like structure all around the perimeter of the Cor-Oids (Figure 3C).

We have shown that a triculture approach is effective in forming cardiac organoids composed of physiological and cardiomyopathic hiPSC-CMs. We observed that the longer the triculture period, the more contact between myocytes and nonmyocytes was established, thus generating more physiological-like cardiac organoids. We conclude that day 14 Cor-Oids were decidedly less physiological than their day 21 counterparts, and we set the latter as the experimental standard for the further generation of organoids containing our hiPSC-CMs.

#### 3.3.2. hiPSC-CM-Based Cardiac Organoids

Following the optimization of the Cor-Oids protocol, in a next step, both the WTSI-CMs and UKK-CM cell lines were used for the generation of organoids. A single cell suspension of either cell type was mixed with both HCMECs and HCFs (3:5:2, 100,000 cells/organoid) to establish organoids representing healthy and HCM cardiac tissue. We named these organoids WTS-Oids and UKK-Oids, respectively.

**Figure 4:**
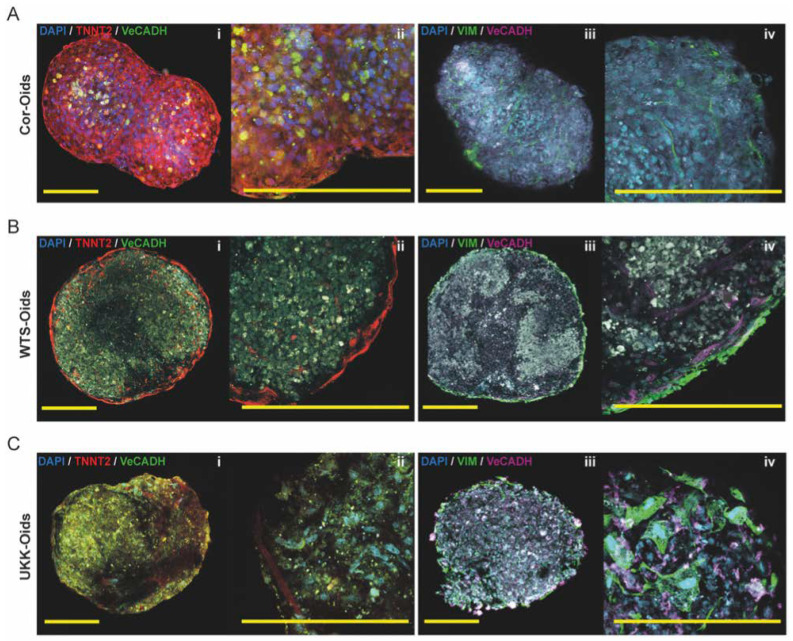
Determining the structural development of cardiac organoids. (**A**–**C**) Immunofluorescence images of Cor-Oids, WTS-Oids and UKK-Oids after 21 days of culture, labelled with cardiac-specific troponin (TNNT2), vascular cadherin (VeCADH) and vimentin (VIM). We identified similar structural patterns (**A**–**C**, iii–iv) in all organoid types, in terms of HCMEC (magenta) and HCF (green) distribution. Different structural development patterns were observed regarding the migration of hiPSC-CMs and the formation of cardiac filament-like structures (red), which were well-defined in Cor-Oids (**A**, i–ii), mostly perimetric in WTS-Oids (**B**, i–ii), and still in formation in UKK-Oids (**C,** i–ii). Scale bars = 200 µm.

The WTS-Oids and UKK-Oids were also maintained for 21 days, and evaluated in terms of cell distribution, migration of CMs towards the organoids’ core and the presence of cardiac-like structures. Immunofluorescence staining of TNNT2, VeCADH and VIM performed on WTS-Oids and UKK-Oids was compared to Cor-Oids at day 21. The HCMEC and HCF cellular distribution were found to be similar in all organoid types (Figure 4A–C, iii–iv). However, several differences were observed regarding the hiPSC-CMs of alternative origin. The CMs in the WTS-Oids were clearly distributed around the perimeter, forming a unique filament-like structure (red staining, Figure 4B, i–ii), while in the UKK-Oids (Figure 4C, i–ii) the formation of a filamentous structure was still in progress. These findings demonstrated that the patient-derived CMs initially form a filamentous structure on the organoids’ perimeter and then migrate to the core, promoting the formation of cardiac filament-like structures (T-tubules and sarcomeres). We speculate that this migration enhances the physiological-like properties of the organoids. However, our findings also indicate that the WTS-Oids were less physiological when compared to the Cor-Oids (Figure 4A, i–ii) and the UKK-Oids appeared to exhibit the least physiological-like properties at 21 days.

These findings may suggest that the expression of the organoids’ physiological-like properties depend on the source of CMs. To assess this further, the first day of contractions and relaxations and the beating time of each organoid type were recorded and compared over 21 days of culture (Table 4). Our data showed that contractions and relaxations (beating cycles) represent a conditioning of the organoids, which enhances the formation of cardiac-like structures and promotes the organoids’ physiological properties over time. Cor-Oids exhibited the longest sustained beating times among the three organoid groups, while WTS-Oids showed intermediate and UKK-Oids the shortest sustained beating times.

#### 3.3.3. Cardiac Organoids Display Patient-Specific Phenotypic Features

**Figure 5:**
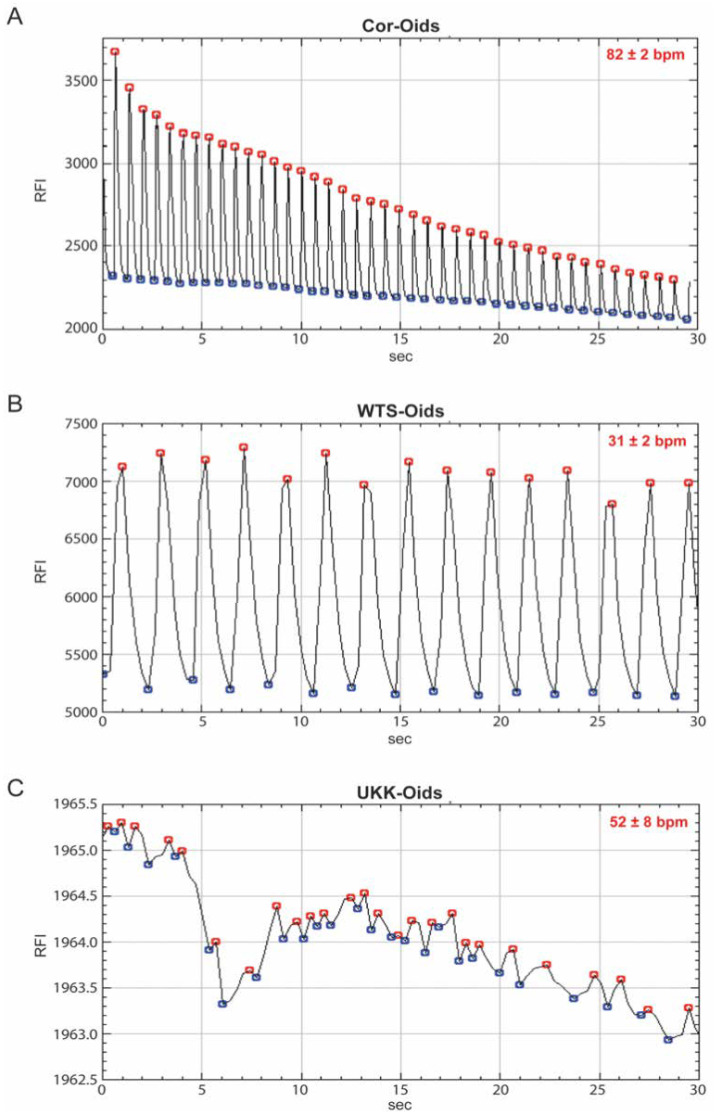
Patient-specific (patho-)physiological features mirrored by cardiac organoids. (**A**–**C**) Analysis of the beating patterns and rates of Cor-Oids, WTS-Oids and UKK-Oids on day 21 **(A)** Cor-Oids showed a regular beating pattern with a beating rate of 82 ± 2 bpm (**B**) WTS-Oids showed a similar regular beating pattern, but a slower beating rate than Cor-Oids (**C**) UKK-Oids presented an arrhythmic beating pattern and a significant variability in their beating rate. RFI = Relative Fluorescence Intensity.

In order to further assess the organoids’ beating patterns and frequencies, a calcium ion binding dye was used to determine their calcium activity after 21 days of culture. As expected, phenotypical differences based on the source of hiPSC-CMs were observed. Cor-Oids were found to have a rhythmically regular beating pattern, with a beating rate of 82 ± 2 bpm (Figure 5A and Supplementary Video S2A) [38]. Interestingly, the beating pattern of WTS-Oids was found to be regular, but with a much slower rate (31 ± 2 bpm) compared to Cor-Oids, which may be due to the fact that the WTSIi020-A-CMs were not sorted for cell purity (Figure 5B and Supplementary Video S2B). Notably, UKK-Oids presented with an irregular beating pattern and a variable beating rate (52 ± 8 bpm), which may correspond to the arrhythmia typically observed in HCM patients (Figure 5C and Supplementary Video S2C) [17]. Our findings confirm that the physiological-like state of the organoids is highly dependent on the culture time.

## 4. Discussion

Cardiomyopathies are a subset of human cardiovascular diseases, which are currently among the leading cause of morbidity and mortality worldwide [5,30]. Although classified into one group, cardiomyopathies present with many differences in terms of disease mechanisms, which renders diagnosis complex and makes it difficult to establish a uniform therapeutic approach [2]. Generally, cardiomyopathies affect the function of the heart due to compromised CM morphology and physiology [39,40]. Unexpected pharmaco-related side effects and toxicities have led to serious outcomes, such as myocardial infarction and the development of chronic heart failure [22,33]. Moreover, the discovery of novel pharmaceutical lead compounds is not only a long and expensive process but is often compromised by drug efficacy and drug safety problems in clinical trials [12]. It is thus of great importance to improve and accelerate the drug discovery process by establishing a human-relevant tissue model, capable of representing human physiological functions in vitro. Organoids may embody the most tangible solution to evaluating drug safety during the early phase of preclinical drug discovery, allowing for the development of risk mitigating strategies by testing patient-specific responses and tailoring more precise treatments [12,39,41]. Indeed, conventional 2D cell culture has significant shortcomings, thereby limiting its relevance in assessing drug safety and efficacy [42]. Some previously established cell culture models using isolated CMs from animals showed their nonrelevance for human translation [40,43]. Models based on human cardiac cell lines were found to be too simplistic and poor in the representation of physiological or pathological states [44]. Previous cardiac organoid models did neither include endothelial cells as a nonmyocyte component in the microtissues, nor contain cardiomyocytes derived from hiPSC lines having a known cardiac disease background [43,45].

Our study aims to elucidate the suitability of cardiac organoids as a novel in vitro model for genetically healthy and hypertrophic cardiomyopathy (HCM) heart conditions. This model could serve as a platform for understanding disease mechanisms as well as designing patient-specific treatments. Making use of the fact that hiPSCs maintain the genotype of the donor, we differentiated CMs from hiPSCs derived from a healthy patient and an HCM patient in order to assess their capacity to represent the corresponding human heart (patho-)physiology [16]. After comparing two differentiation protocols for robustness and efficiency, the protocol developed by Burridge et al. was selected to derive healthy (WTSI-CMs) and HCM (UKK-CMs) CMs from hiPSCs [21]. We found this protocol to be the most economical and efficient differentiation protocol for use in the academic lab. The differentiated cells were characterized by flow cytometry, with the majority of the differentiated cells (day 15) proving positive for TNNT2, as confirmed by calculating the median fluorescence intensities (MFI) of the light emitted by TNNT2^+^ populations. Further characterization was performed examining the gene expression patterns on both (WTSIi- and UKKi-derived cells) day 15 TNNT2^+^ populations, showing downregulation of pluripotency (NANOG, OCT4, SOX2) and carcinogenicity (KLF4, MYC) markers, which demonstrates high differentiation efficiency and absence of oncogenicity. Cardiac markers (NKX2.5, TNNT2, MYH6 and MYH7) were found upregulated, which suggests the formation of advanced cardiac structures. However, the high expression of NKX2.5 and MYH6 indicate that both day 15 WTSI-CMs and UKK-CMs were still rather immature cardiomyocytes [16]. Once the differentiation protocol was established, WTSI-CMs and UKK-CMs were used for the generation of cardiac organoids, which we labelled WTS-Oids and UKK-Oids, respectively. Our aim in performing triculture cell seeding to establish cardiac organoids is to provide more physiological-like function and structure in an in vitro model. The triculture cell ratio — consisting of three parts cardiomyocytes, five parts endothelial cells and two parts fibroblasts — was chosen to resemble the corresponding cell ratio in the adult human heart [28]. This ratio is, however, highly variable and subject to change, according to the different developmental stages of the human heart.

To prevent overgrowth of the endothelial cells and fibroblasts, we used a cardiomyocyte maintenance medium during the triculture organoids culture. In the myocardium, endothelial cells are known to regulate the contractile state of CMs through autocrine and paracrine signaling molecules, while fibroblasts play an essential role in the maintenance and remodeling of the 3D structure through the synthesis of extracellular matrix (ECM) [12,22,28].Therefore, we used a triculture approach of hiPSC-CMs, HMCECs and HCFs for the generation of highly contractile cardiac organoids, with the goal of mimicking the cardiac microenvironment as closely as possible in 3D. As a parallel comparison, commercially available cardiomyocytes Cor.4U-CMs were used to generate what we defined as standard organoids (Cor-Oids). The Cor-Oids exhibited more physiological-like properties than the hiPSC-derived organoids after 21 days in culture, exhibiting sustained beating, which may indicate that extended myocyte and nonmyocyte cell-to-cell contact may promote the formation of more physiological cardiac-like structures.

Cell distribution, CM migration, appearance of cardiac structures and beating patterns as well as beating rates of WTS-Oids and UKK-Oids were compared to the Cor-Oids to evaluate the functional features and the physiological-like properties at the same timepoint (day 21). We found that CM distribution is dependent on organoid culture time to allow for the migration of CMs towards the core of the 3D microenvironment. This migration promotes the interaction of CMs with nonmyocytes, leading to the formation of filamentous-like structures, which most likely represent T-tubules and sarcomeres. Moreover, we observed that the appearance of the beating cycle is directly related to the formation of T-tubules and sarcomeres, which, in turn, is strictly associated with the organoids’ sustained beating time. This may explain why WTS-Oids and UKK-Oids show weaker beating, and hence less physiological-like properties, when compared to Cor-Oids. Finally, we observed that WTS-Oids present a rhythmic regular beating pattern while UKK-Oids present with arrhythmia, which is typical for HCM patients. This suggests that organoids retain the phenotype of the hiPSC donor.

Taken together, the proposed in vitro organoid model may represent a physiological-like human heart mini-tissue, which retains the geno- and phenotypical characteristics of the corresponding donor tissue. Therefore, our organoids may represent a robust model system, suitable for medium- to large-scale production, for studying and modeling cardiac disease. Feasibility assays for producing organoids in a more size-controlled, automated and high-throughput fashion is envisioned. It should be noted that this system cannot entirely replace animal models due to the bypassing of critical composite features of human diseases, such as complex sensory and feedback systems.

We acknowledge that our study has several limitations. Firstly, the question that has to be addressed in more detail in the future is the maturation of hiPSC-derived CMs. A recent study suggests that multicellular contact over prolonged culture periods could enhance maturation [46]. Secondly, the observed differences between the hiPSC lines and the organoids need to be investigated further using a larger quantity of organoids. The mutation in the HCM hiPSC-CMs should be confirmed by total DNA sequencing. Thirdly, it is understood that modeling cardiomyopathy using the triculture approach does not account for further influencing factors, such as immunological responses and additional cardiac cell fractions (e.g., CM subpopulations like Purkinje fibers). Immune cells could ideally be cultivated on the outer layer of the organoids to monitor immunological responses. It would be insightful to elucidate immune cell migration patterns in the rather loose-structured, cardiomyopathy-representing organoids. Finally, it would be ideal to derive all organoid cell fractions from the identical parental hiPSC line for the triculture approach. There exist established protocols to derive hiPSCs into endothelial cells and cardiac fibroblast [47,48]. We suggest that the recapitulation of cardiomyopathic properties in such organoids could be even more pronounced, especially in diseases where the pathophysiology does not solely stem from cardiomyocytes pathology but also from nonmyocyte components.

## 5. Conclusions

In the present study, we demonstrate the differentiation of isogenically comparable hiPSC lines into cardiomyocytes from a healthy human donor and a patient with hypertrophic cardiomyopathy for the generation of human cardiac organoids. To mimic adult human heart tissue, healthy and HCM-associated hiPSC-CMs were combined with HCMECs and HCFs to generate highly contractile triculture organoids. These organoids are capable of representing significant phenotypical features of the healthy and hypertrophic cardiomyopathic human heart in vitro.

Obtaining hiPSCs, reprogrammed from patient-derived somatic cells, offers not only the possibility of differentiating all cell types involved in the generation of highly contractile cardiac organoids but may open up new perspectives for the development of safer and more efficacious patient-tailored therapeutic strategies. Our novel cardiac organoids may, therefore, represent a valuable platform for high-throughput testing and validation of novel therapeutic agents to treat cardiomyopathy.

## Figures and Tables

**Table 1:** Human induced pluripotent stem cell (hiPSC) lines obtained from the European Bank of Induced Pluripotent Stem Cells (EBiSC).

hiPSC Line	Gender	Age	Race	Remarks
WTSIi020-A	Female	40–44	Caucasian	Derived from healthy donor
UKKi025-A	Female	40–44	Caucasian	Derived from hypertrophic cardiomyopathy (HCM) donor with MYH7 mutation. The mutation is a heterozygous missense mutation of the β-myosin heavy chain MYH7 gene located in exon 19, at chromosome location 14q11.2. There is a change of the single nucleotide sequence at position 2156 from G to A which causes the exchange of a nonpolar amino acid for a positively charged one (Arginine changed into Glutamine) in codon 719.

**Table 2:** Taqman gene expression assays used to prove the efficiency of Chemically-Defined Medium 3 (CDM3) differentiation protocol through real-time quantitative polymerase chain reaction (RT-qPCR).

Gene Category	Symbol	Gene Name	Taqman Primer ID
Housekeeping	18S	Eukaryotic 18S rRNA	Hs99999901_s1
Pluripotency	NANOG	Nanog Homeobox Protein	Hs02387400_g1
	OCT4 (POU5F1)	Octamer-binding Transcription Factor 4	Hs00999632_g1
	SOX2	SRY (Sex determining Region Y)-box 2	Hs01053049_s1
	KLF4	Kruppel Like Factor 4	Hs00359936_m1
	MYC	V-Myc Avian Myelocytomatosis Viral Oncogene Homolog	Hs00153408_m1
Cardiac Development	NKX2.5	NK2 Homeobox 5	Hs00231763_m1
Cardiac Structure	TNNT2	Cardiac-specific Troponin	Hs00165960_m1
	MYH6	Myosin Heavy Chain 6	Hs00411908_m1
	MYH7	Myosin Heavy Chain 7	Hs01110632_m1

**Table 3:** Differentiation protocol comparison. The STEMdiff and CDM3 protocols were compared to differentiate cardiomyocytes (CMs) using two hiPSC-lines, one from a healthy patient (WTSIi020-A) and another from a hypertrophic cardiomyopathy patient (UKKi025-A). In terms of cell quality, the CDM3 protocol proved to be better for the differentiation of WTSIi020-A and UKK025-A into CMs.

		WTSIi020-A			UKKi025-A		
Batch No.	Protocol	Passage	Confluence	CMs Quality	Passage	Confluence	CMs Quality
1st	STEMdiff	38	95%	Low	42	95%	Mid
2nd	STEMdiff	39	95%	Differentiation Failed	41	95%	Mid
3rd	STEMdiff	39	95%	×	41	95%	×
	CDM3	39	85%	Mid	41	85%	High
4th	CDM3	40	85%	High	41	90%	High
5th	CDM3	42	90%	High	42	95%	High

**Table 4:** The organization of organoids depends on their sustained beating time.

Organoid Type	Visible Contractions and Relaxations	Sustained Beating Time
Cor-Oids	Day 2,3	~3 Weeks
WTS-Oids	Day 7,8	~2 Weeks
UKK-Oids	Day 12–15	~1 Week

Over 21 days of culture, the day when contractions and relaxations were recognized and the sustained beating time of each organoid type were found to be key points for determining their structural organization. Indeed, contractions and relaxations over time stimulate the formation of defined cardiac-like structures.

## Supplementary Materials

The following are available online at https://www.mdpi.com/2073-4409/9/7/1733/s1. Beating videos of (S1A) WTSIi020-A CMs and (S2B) UKKi025-A CMs; Calcium activity videos of the (S2A) Cor-Oids, (S2B) WTS-Oids and (S2C) UKK-Oids, obtained using a wide field microscope.

## Abbreviations

CMs	cardiomyocytes
Cor-Oids	Cor.4U-based organoids
ECM	extracellular Matrix
ECs	endothelial cells
HCFs	human cardiac fibroblasts
HCM	hypertrophic cardiomyopathy
HCMECs	human cardiac microvascular ECs
hiPSCs	human induced pluripotent stem cells
hiPSC-CMs	hiPSC-derived CMs
KLF4	kruppel like factor 4
MFI	median florescence intensity
MYC	V-Myc avian myelocytomatosis viral oncogene homolog
MYH6	myosin heavy chain 6
MYH7	myosin heavy chain 7
NANOG	nanog homeobox protein
NKX2.5	NK2 homeobox 5
OCT4	octamer-binding transcription factor 4
RT-qPCR	real time quantitative PCR
SOX2	SRY (sex determining region Y)-box
TNNT2	cardiac-specific troponin
UKK-CMs	UKKi025-A derived CMs
UKK-Oids	UKK-CMs based organoids
VeCADH	vascular cadherin
VIM	vimentin
WTSI-CMs	WTSIi020-A derived CMs
WTS-Oids	WTSI-CMs based organoids
